# A Survey among Dog and Cat Owners on Pet Food Storage and Preservation in the Households

**DOI:** 10.3390/ani11020273

**Published:** 2021-01-21

**Authors:** Giada Morelli, Davide Stefanutti, Rebecca Ricci

**Affiliations:** Department of Animal Medicine, Production and Health, University of Padua, Viale dell’Università 16, 35020 Legnaro, Italy; davide.stefanutti@phd.unipd.it (D.S.); rebecca.ricci@unipd.it (R.R.)

**Keywords:** dog, cat, pet food, storage, survey

## Abstract

**Simple Summary:**

Proper dog and cat food preservation is fundamental to ensure the best quality of the diet right up to the moment of feeding, given that nutritional value, safety and sensory properties can be irreparably damaged by environment and time. To this end, a survey was conducted to explore how pet owners store both commercial and home-prepared diets. On the whole, despite the wide variability of practices adopted, most of the 2221 respondents implemented good storage management. Room temperature was the most overlooked parameter during storage, and this may be a cause of concern because exposure to warmth can enhance rancidity, especially in diet formulations rich in fats and oils. For this reason, veterinarians should provide precise instructions on storing perishable ingredients to those who feed home-prepared diets. Moreover, respondents of older generations appeared to distrust the use of preservatives in commercial pet foods and often deemed their inclusion harmful and unnecessary. Pet food manufacturers should discourage skepticism of the additives crucial to long-term commercial pet food conservation through better communication.

**Abstract:**

Background: Pet food storage plays a crucial role in maintaining the nutritional and sensory properties of purchased products over time. Methods: An online survey was developed to collect data regarding owners’ storage habits for both commercial and home-made diets. Results: The questionnaire was completed by 1545 dog owners and 676 cat owners. Pet and owner age played roles in the choice of the type of diet (commercial vs. home-cooked vs. raw meat-based) adopted. Kibble feeders (75.7%) usually bought one (50.1%) or two (24.6%) packages at a time, and most pets (64.4%) took a minimum four weeks to consume an entire bag. Almost half of the owners (43.5%) used a container to store pet food (plastic bins for 79.5%). Pet food was commonly stored in the kitchen (45.1%) and not exposed to direct light (94.5%); 23.6% of the kibble feeders said it might be exposed to high temperatures. Most commercial pet food feeders (67.3%) considered preservatives a potential health risk for pets. Among homemade diet feeders, 38.6% stored fish oil at room temperature. Conclusions: Pet owners should be educated in proper food storage management when receiving feeding instructions from veterinarians. More comprehensive information on the nature and importance of additives in pet food should be promoted by manufacturers.

## 1. Introduction

Like food for human consumption, pet food is closely regulated to ensure the highest standards of hygiene, safety and quality. Along with this objective, European pet food manufacturers must abide by numerous regulations that involve the entire production process, from the selection of raw materials to the sale of finished products [1]. The ultimate quality and safety of pet foods are determined by all the measures taken throughout their production, distribution and use, however. Critical hazards from the first steps in the supply chain to the final handling before consumption can be identified, and this makes also consumers responsible for preserving the nutritional properties and safety of the food products together with retailers, distributors and manufacturers [2,3].

The most overlooked players in ensuring pet food quality and safety are the owners, whose domestic storage and handling habits have been scarcely considered in literary evidence until now. Even if dry and wet commercial pet foods are sold as nutritionally adequate and safe, improper management after purchase can worsen their quality [4] and this may eventually place both people and their animals at risk of food-related issues [3]. This applies also to the fresh human-grade ingredients used in home-prepared diets, which may cause a decline in the nutrient and microbial profile of the diet if not managed and handled properly. Since the consumption of altered foods can negatively affect animal wellness and health [4,5], care must be taken to preserve their sensory profile, nutritional value and microbiological safety until administration to the pet.

Proper pet food storage is paramount to blocking or delaying the development of degenerative chemical processes and the growth of spoilage microorganisms which lead to its alteration [5,6]. Whether intended as commercial product or home-prepared meal, together with the length of time for which it is suited for consumption, the diet’s nutritional characteristics mainly define the extent of chemical and microbiological risks to which it is exposed during storage. Due to the high lipid content and the long shelf life of kibble, lipid oxidation poses a major spoilage risk for dry pet foods [3,4,5]. Lipid oxidation is a time-dependent degenerative chemical phenomenon that entails major sensory alterations and a decline of the food’s nutritional value [3], and eventually leads to the formation of compounds with mutagenic and genotoxic potential [6,7,8,9]. Whereas on the other hand high extrusion temperatures and low moisture content decrease the risk of microbial contamination in kibbles [10], such finished dry pet food products offer a suitable substrate for *Salmonella* spp. survival in the long term [2,3,4,5,6,7,8,9,10,11]. Similarly, wet pet foods packaged in cans, trays and pouches undergo heat processing to achieve commercial sterility by destroying heat-sensitive pathogens; for this reason, these products should not contain pathogens at the time of opening.

Home-prepared diets require the use of fresh perishable ingredients, among which meat is the most fragile owing to its chemical composition that favors microbial contamination and growth during storage [12]. However, home-prepared diets are usually consumed immediately after preparation; thus, their relatively short storage time makes the threat posed by spoilage processes less concerning. Moreover, domestic cooking is an effective method to significantly reduce the microbial contamination of the ingredients. On the contrary, the microbiological risk is definitely higher for raw meat-based diets (RMBDs), which have been gaining popularity in recent years among dog and cat owners [13], since raw meat might be contaminated by foodborne pathogens [14]. Neither should lipid oxidation be underestimated for cooked or raw home-prepared diets, due to the widespread use of oils rich in polyunsaturated fats (PUFAs; e.g., fish oil) which oxidize easily if not stored properly [15].

While gathering information on the type of diet most commonly adopted among the dog and cat owners interviewed, the main goals of this study were to acquire knowledge on the most popular pet food domestic preservation methods and to point out any criticality in the storage management of both commercial and home-prepared diets. The behavior of the owners during pet food purchase and their attitudes to diet preparation and administration were also investigated.

## 2. Materials and Methods

### 2.1. Participant Recruitment and Survey Design

A multiple-choice online survey (Supplementary file S1) in Italian language was created using Google Forms©. The questionnaire was piloted among the authors before its publication and the results of this test were not considered in the study. The open survey was shared on a social media (Facebook©) for 60 days between December 2018 and January 2019; it was actively promoted to a number of pet enthusiasts, owners and breeders’ groups. No reference to a specific diet was included in the title of the questionnaire (“Preservation of foods for dogs and cats”) so that any owner, regardless of the type of diet adopted for their pet, could volunteer to participate in the study.

The survey began with multiple choice questions (MCQs) that collected information on demographic data (i.e., gender, age, place of residence, job association with animal care), pet species (i.e., dog or cat; owners of many animals were asked to fill out the questionnaire by referring to one single pet), pet signalment (i.e., breed, gender, age, size, body weight, body condition according to the owner) and type of diet administered to the pet (i.e., commercial dry diet, commercial wet diet, home-cooked diet (HCD), RMBD). Participants were then interviewed on the domestic management of pet food depending on the selected type of diet. Those who used kibbles on a daily basis, whether alone or in combination with canned pet foods or homemade foods, were invited to answer the dry food-related MCQs; those who used commercial wet food alone or in combination with homemade foods were asked to answer the wet pet food-related MCQs. For both groups (dry food-centered and wet food-centered), an initial set of questions investigated owners’ purchasing and pet feeding habits; a second set of MCQs collected information on the characteristics of the diet, and a third set of questions examined storage habits. Lastly, three additional Likert scale questions were included with the aim of collecting opinions on the use of preservatives in commercial pet food. For those who used a HCD or a RMBD only, a first set of MCQs aimed at investigating diet formulation, while a second one aimed at studying meat and fish preservation practices; a third and last set of MCQs investigated the use of fish oils and vegetable oils and their storage conditions.

In total, the survey contained 118 questions, but the number of questions per participant varied from 35 to 48 depending on the type of diet selected.

### 2.2. Data Analysis

The data collected from the survey were transferred to a spreadsheet (Excel, Microsoft) and underwent descriptive analysis. Quantitative and qualitative data were reported as frequency (n/N) and percentage (%). Data expressed as percentages were compared among categories using either the Chi-square test or the Fisher exact test for small samples (SAS version 9.4). Statistical significance was set at *p* < 0.05.

## 3. Results

### 3.1. Survey Participants

A total of 2221 persons answered the survey, 1545 (69.6%) of whom were dog owners and 676 (30.4%) were cat owners. Most interviewees were women (87.4%, 1940/2221) and were 18–34 years old (40.3%, 894/2221). Participants were asked whether their job dealt with animal care: 4.6% (103/2221) were dog trainers, 2.3% (50/2221) breeders, 2.0% (44/2221) veterinarians, 1.6% (35/2221) veterinary medicine students and 0.9% (21/2221) pet groomers. Pet owner demographics are presented in Table 1.

### 3.2. Canine Population

Out of a total of 1545 dogs, males and females were equally represented (51.2% and 48.8%, respectively). The percentage of neutered animals varied widely depending on gender: most males were intact (78.4%, 619/790), while 56.2% (424/755) of females were spayed. Roughly, one in five dogs (302/1545) was mongrel, and among 115 different breeds the most representative was Border Collie (5.8%, 90/1545), followed by Weimaraner and Labrador Retriever. As regards the age of the dogs, 58 responses were excluded because an unlikely date of birth was reported. Most dogs were aged between 1 and 7 years (65.1%, 968/1487); the mean age was 46 months for purebred dogs and 64 for mongrels, accounting for an overall mean of 50 ± 41 months. The majority of the dogs were medium size (10–24 kg, 41.7%), while 29.6% were large size (25–45 kg), 24.1% small size (<25 kg) and 4.7% giant size (>45 kg). Most owners considered the weight of their dogs to be ideal (85.2%), while 10.1% of the dogs were reported to be overweight, 4.6% underweight and none obese. Table 2 provides a summary of dog demographics.

### 3.3. Feline Population

Out of a total of 676 cats, 367 were males (54.3%) and 309 females (46.7%). There was no evident difference in the rate of neutered cats between males (87.2%) and females (87.7%).

Regarding age, six responses were excluded because an unlikely date of birth was reported. Evaluation of the 670 valid answers gave the following distribution in age groups: 6.1% of cats were less than 6 months old, 23.5% had an age between 6 months and 2 years, 57.3% were between 2 and 10 years old, and the remaining 13.1% were more than 10 years old. Most cats (*n* = 517) were European Shorthair (no pedigree), and the remaining 159 cats belonged to 23 different breeds. Among these, the most representative was Maine Coon, followed by Scottish Fold and Norwegian Forest Cat breeds.

Most owners considered the weight of their cats to be ideal (71.3%), while 23.4% of the cats were reported to be overweight, 4.0% underweight and 1.3% obese. Table 3 provides a summary of cat demographics.

### 3.4. Types of Diets and Demographics

The majority of pet owners (75.7%, 1682/2221) fed their pets kibble. Among them, 37.7% (634/1682) relied exclusively on dry pet food, while 35.3% (594/1682) mixed dry and wet pet food, 14.0% (236/1682) mixed kibble and homemade diet, and 13.0% (218/1682) mixed dry and wet pet food and homemade diet.

The remaining participants (24.3%, 539/2221) who did not feed any kibble were distributed as follows: commercial wet pet food only: 1.8%, 39/2221; commercial wet pet food mixed with homemade diet: 1.9%, 42/2221; HCD: 10.0%, 222/2221 (214 dog owners and 8 cat owners), and RMBD: 10.6%, 236/2221 (207 dog owners and 29 cat owners).

The distribution of the diets based on demographics is shown in Table 4.

The Chi Square test highlighted a significant relationship (*p* = 0.0004) between the dog’s diet and the age of the owner. The incidence of feeding dry food (either as single or additional component of the diet) was higher among the under-35s (75.4%, 492/652) but lower among people between 35 and 64 years (64.3%, 545/847) and among the over-65s (59.6%, 28/47). Conversely, feeding a HCD was more common among the older sections of the population, especially the over-65s (29.7%, 14/47), where it reached a much higher percentage than in other age groups. With the exception of the over-65s (8.5%, 4/47), RMBDs were preferred equally by all sections of the population. No significant relationship emerged between the age of the owner and the cat’s diet instead.

Another significant item of evidence (*p* < 0.0001) was found between type of diet and dog’s age: kibbles were preferred for feeding puppies rather than adult and senior dogs.

As regards the relationship between age and diet in cats, canned food-based diets were more popular among senior cats (12.5%, 11/88) and kittens (4.9%, 2/41), while among junior subjects and adults the prevalence of this diet did not reach 2% (*p* = 0.0004). HCDs and RMBDs were quite unpopular among cat population at any life stage.

### 3.5. Dry and Wet Pet Food-Based Diet: Selection, Management and Purchase

Questions on dry pet food were answered by 1682 people but due to a technical problem, the 236 owners feeding their pet kibbles mixed with home-prepared food could not answer the 14 questions related to selection, management and purchase (i.e., questions 22 to 35, Sections 5 and 6 of the survey); therefore, only 1446 answers were taken into consideration. The majority of the owners who gave their animals kibble as single or additional component of the diet habitually fed their pet twice a day (48.4%, 700/1446); 22.1% (320/1446) preferred *ad libitum* feeding, 19.1% (276/1446) administered three meals per day, 5.4% (78/1446) more than three meals per day, and the remaining 5.0% (72/1446) a single daily meal.

The quantity of dry food administered was mainly based on personal experience (42.0%, 607/1446) and packaging indications (33.5%, 484/1446), while one owner out of four (24.5%, 355/1446) relied on the advice of the veterinarian. Doses were measured on a kitchen scale (29.6%, 428/1446), by eye (28.4%, 411/1446) or using a measuring cup (28.0%, 405/1446); 14.0% (202/1446) of the participants did not consider measuring dry pet food at all.

The pet shop was selected as the preferred point of dry pet food purchase (60.7%, 877/1446), while large retailers (i.e., supermarkets, convenience stores) were chosen by 10.7% (155/1446) of the participants; other common purchase methods were internet shopping (25.4%, 368/1446) and door-to-door selling (3.2%, 46/1446).

Half of the owners purchasing dry pet food for their dog or cat habitually bought one pack at a time (50.1%, 724/1446), while the others used bought at least two (24.6%, 356/1446), or three or more (25.3%, 366/1446). The most purchased kibble bag sizes weighed more than 11 kg (36.1%, 522/1446) and 1–2 kg (23.6%, 341/1446); other common bag sizes weighed less than 1 kg (17.3%, 250/1446), 3–6 kg (13.7%, 198/1446) and 7–10 kg (7.9%, 115/1446). A significant relationship (*p* < 0.0001) was found between kibble bag size and pet species, as bags weighing less than 6 kg were preferred by cat owners (63.4%, 500/789) while those weighing over 6 kg were much more often purchased by dog owners (87.3%, 556/637). A significant relationship (*p* < 0.0001) was also observed between kibble bag size and dog size, as bags weighing less than 6 kg were preferred for small size dogs (52.9%, 153/289) while those weighing over 6 kg were mainly purchased for medium and maxi size dogs (82.7%, 460/556). Giant dog owners purchased only bags that weighed between 11 and 15 kg (*n* = 29) or more than 15 kg (*n* = 13).

Twenty respondents (1.4%) reported purchasing bulk kibble sold in pet stores, and most were cat owners (70.0%, 14/20). Most animals took at least four weeks to consume an entire dry food pack (64.4%, 931/1446) while some required less than two weeks (22.5%, 326/1446) or two to four weeks (13.1%, 189/1446). The Chi Square test highlighted a significant relationship (*p* < 0.0001) between time taken to consume a whole pet food bag and animal species. Most of the dogs took one month or more to consume a package (71.8%, 611/851) while in cats this percentage was considerably lower (53.8%, 320/595). As regards differences among the dog sizes, no clear trend was found.

As for the dry food type currently fed, standard maintenance pet food was the most popular (43.3%, 626/1446); 20.5% of the owners (296/1446) reported using a grain-free product, 14.2% a calorie-restricted product (205/1446) and 13.1% a dietetic product (189/1446), while vegan and vegetarian formulations were chosen by 0.4% of the interviewees (6/1446). The four most common dietetic pet foods were those formulated for the management of gastrointestinal diseases (4.8%, 70/1446), kidney diseases (3.7%, 53/1446), obesity (3.1%, 45/1446) and dermatological disorders (2.8%, 41/1446). Single-protein formulations were chosen by 39.9% of the respondents (578/1446), while one in four owners (24.7%, 357/1446) could not say whether the food in use included limited ingredients or not.

The choice of dry food type was mainly influenced by the owner’s personal experience (27.2%, 393/1446), the veterinarian’s advice (23.8%, 345/1446) and information available on internet (17.2%, 249/1446). Some people followed advice from breeders (8.4%, 121/1446), shop assistants (7.3%, 105/1446) and friends or relatives (3.9%, 56/1446); the remaining 12.2% (176/1446) stated other reasons.

Questions about wet pet food were answered by 81 people, including 60 dog owners and 21 cat owners. Most animals fed canned foods were usually given two (60.5%, 49/81) or more (29.6%, 24/81) meals per day, while 6.2% (5/81) were given a single daily meal and 3.7% (3/81) were fed *ad libitum*. The daily ration was determined by the owner’s expertise (56.8%, 46/81), indications on the packaging (23.5%, 19/81) or the veterinarian’s advice (19.8%, 16/81). Wet foods were mainly purchased at pet shops (46.9%, 38/81) and online shops (35.8%, 29/81), while large retailers were preferred to lesser extent (17.3%, 14/81).

Regular maintenance (58%, 47/81) and grain-free (23.5%, 19/81) were the most common wet food types; among dietetic products (9.9%, 8/81), those for kidney and gastrointestinal diseases prevailed (*n* = 4 each), followed by formulations for diabetic subjects (*n* = 2). Calorie-restricted products were purchased by 3.7% (3/81) while the remaining 4.9% bought other types of wet food (4/81). Single-protein formulations were chosen by 38.3% of the respondents (31/81, while eleven owners (13.6%) could not say whether the food in use included limited ingredients or not.

Personal experience (42.0%, 34/81) and internet searches (28.4%, 23/81) were the main determining factors in product choice while 17.3% (14/81) selected the food based on other reasons, such as other people’s advice or the preference shown by the pet. The remaining 12.3% (10/81) purchased the products recommended by the veterinarian.

The most common packaging types were rigid cans (58.0%, 47/81), aluminum trays (25.9%, 21/81) and pouches (16.1%, 13/81). The most purchased formats weighed between 100 and 400 g (50.6%, 41/81), or more than 400 g but less than 1 kg (24.7%, 20/81); 19.8% (16/81) of the owners reported using formats weighing less than 100 g while 4.9% (4/81) bought packages weighing more than 1 kg.

### 3.6. Dry and Wet Pet Food-Based Diet: Storage

As for the storage of dry pet food, more than half of the kibble feeders reported keeping the product in its original package (56.5%, 951/1682), while others moved it partially (23.0%, 387/1682) or totally (20.5%, 344/1682) to another container. The most popular alternative containers were bins made of plastic (79.5%, 581/731) or tin (14.8%, 108/731), and a minority of the interviewees (5.7%, 42/731) said they used automatic pet feeders, glass containers or nylon bags. The pet food package in use was generally closed using clothespins, adhesive tape or rubber bands (43.8%, 586/1338) or the resealable zip lock included (40.1%, 536/1338); 1.8% (25/1338) used alternative methods (e.g., tinfoil). Some owners either just rolled the edges of the bag (11.0%, 147/1338) or did not close it at all (3.3%, 44/1338). The original packaging was a cardboard box in 24.8% of cases (417/1682).

Kibbles were stored inside the house, more precisely in the kitchen (45.1%, 758/1682), in a closet (31.8%, 535/1682) or in other rooms (16.0%, 269/1682); conversely, some interviewees stored kibbles outside the house in a closed environment (e.g., the garage; 6.7%, 113/1682) or outdoors (0.4%, 7/1682). The pet food bag was commonly placed inside a cabinet (52.7%, 885/1682) or another container (16.9%, 285/1682) or else kept on the ground by 30.4% of the respondents (512/1682; 320 of which in direct contact with the floor).

According to 94.5% of the owners (1590/1682), the pet food was not exposed to light. Also, the pet food was not positioned near heat sources (92.2%, 1552/1682), and the temperature of the room where it was stored allegedly never exceeded 30 °C (76.4%, 1286/1682).

One in ten interviewees (10.2%, 171/1682) reported perceiving anomalous or unpleasant smells at least once when opening an unexpired package, while 6.8% (115/1682) stated that malodors developed during the regular use of the product. Insects or larvae were found by 4.2% (70/1682) respondents at the first opening of an unexpired package, and by 3.0% (51/1682) during its use.

Four out of ten owners (39.1%, 658/1682) believed kibbles could be consumed for up to two months after opening the package, 33.4% (562/1682) for up to one month and 27.5% (462/1682) for more than two months after opening. A minority of interviewees (10.6%, 179/1682) reported that they never looked at the expiration date on the package, and another 7.2% (121/1682) declared they gave expired kibble to their pets.

Over one in three owners (34.4%, 579/1682) deemed their pets to be more attracted to kibbles from newly-opened packages rather than those from long-opened packages; this percentage drops to 28% when considering dogs and rises to 46% for cats. Another 19.0% (319/1682) was unable to determine whether the pet showed any preference, and according to the remaining 46.5% (784/1682) there was no such difference.

As for the management of leftover kibbles, 52.1% (876/1682) of the owners left them in the bowl to be consumed later by the pet, while 22.4% (377/1682) tossed them in the trash; the others stored them out of the bowl at room temperature (21.9%, 369/1682) or in the refrigerator (3.6%, 60/1682) until the following meal.

Wet pet food storage was not an issue for one third of the interviewees (30.9%, 25/81) since they used single-dose packs. Among the remaining 56 people, 91.1% (51/56) kept the unfinished packages in the refrigerator; the refrigerated food was usually warmed up before consumption by the pet (72.6%, 37/51). The majority of owners (85.7%, 48/56) stored the unfinished packages after closing them with different methods (e.g., plastic wrap, tinfoil, clothespins, plastic bags); six owners (10.7%) moved the remaining food to an airtight container and two (3.6%) did not close the package at all. Among those who did not use single-dose packs, 71.4% (40/56) stored the unfinished packages for up to one day, 14.3% (8/56) for up to two days and 14.3% (8/56) for more than two days. According to 82.7% (67/81) of the people answering the questions on wet pet foods, these products were never exposed to temperatures above 30 °C.

As for the management of leftover wet food, 45.7% (37/81) tossed it in the trash, 29.6% (24/81) left it in the bowl and 24.7% (20/81) stored and administered it with the following meal.

### 3.7. Owners’ Opinions on the Use of Preservatives in Commercial Pet Food

**Figure 1:**
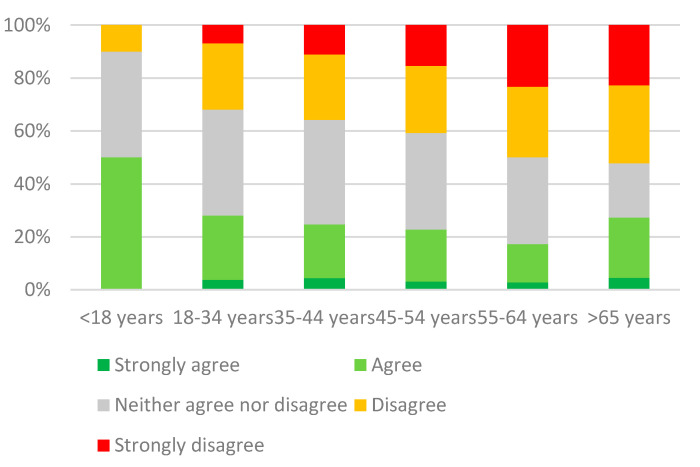
Degree of agreement for each age bracket with the statement: “the use of preservatives in pet food is necessary for optimal preservation”.

Answers to Likert scale questions showed that 25.2% (444/1763) of the pet owners who used commercial pet foods trusted the use of preservatives in these products as necessary for optimal preservation, while 36.9% (650/1763) disagreed with this claim and 37.9% (669/1763) did not take a clear stance on the statement (Figure 1). A significant relationship (*p* < 0.0001) emerged between this affirmation and the respondents’ age, as the percentage of those who did not think preservatives were necessary was significantly higher among participants more than 45 years old (44.5%, 258/580) than among those younger (33.1%, 392/1183).

**Figure 2:**
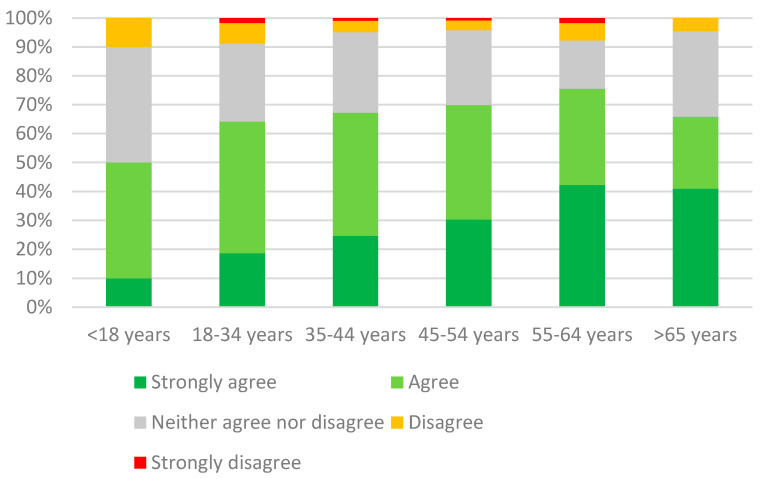
Degree of agreement for each age bracket with the statement: “the presence of preservatives in pet food could be harmful to my pet’s health”.

A small minority of the respondents (6.7%, 118/1763) believed that preservatives were not harmful to the pet’s health, while 67.3% (1185/1763) considered preservatives a potential health risk for pets; 26.1% (460/1763) could neither agree nor disagree with this statement (Figure 2). Again, a significant relationship between the statement “preservatives can be harmful to the pet’s health” and the age of the respondents was found (*p* < 0.0001): whereas only one in five of participants younger than 45 (20.8%, 246/1183) firmly agreed, the belief was shared by more than one out of three older owners (34.8%, 202/580). Lastly, 69.1% of the latter group (1217/1763) believed preservatives in pet foods to consist mostly or entirely of chemicals, while only 4.8% (85/1763) thought they were mainly of natural origin; 26.1% (461/1763) considered them half chemical, half natural.

### 3.8. Home-Cooked Diet: Management, Purchase and Storage

A total of 222 people filled in this section of the survey. The main reason why these owners fed their pets a HCD was their lack of trust in commercial pet food (44.6%, 99/222); otherwise, this type of diet was chosen in support of the pet’s health issues (36.9%, 82/222), overcoming the pet’s palatability issues (e.g., the animal refused to eat commercial pet food, 12.6%, 28/222) or by following the veterinarian’s advice (5.9%, 13/222).

Over one third of the owners (36.5%, 81/222) trusted a veterinary nutritionist for the formulation of the diet, while 24.8% (55/222) relied on their veterinarian and 14.0% (31/222) turned to an online nutritionist consultant. The others relied on their own expertise (11.7%, 26/222), information available on internet (6.8%, 15/222) or specific books (6.3%, 14/222).

As regards the feeding schedule, most dogs were fed twice a day (75.2%, 167/222), while 5.9% (13/222) of the owners administered one meal per day and 18.9% (42/222) three or more. The quantity of food provided was generally weighed with a kitchen scale (78.8%, 175/222); alternatively, 18.0% (40/222) of the daily rations were defined by eye and 3.2% (7/222) were not measured at all. Fish was included in the diet by 79.3% of the HCD-feeders (176/222). Solid fats were used by 22.5% of the owners (50/222); those most commonly used were butter (6.8%, 15/222) and lard (5.4%, 12/222). Half of the interviewees (52.3%, 116/222) bought the meat or fish at the supermarket, 35.1% (78/222) from the butcher or fishmonger and 1.4% (3/222) on internet; 11.3% (25/222) patronized all the above.

Most respondents (80.6%, 179/222) bought fresh meat and fish to be frozen at home, while the minority purchased and stored only fresh ingredients (16.2%, 36/222), or bought them already frozen (3.2%, 7/222).

Except for those who used only fresh ingredients, the meat was generally stored in the freezer for no longer than one month (81.2%, 151/186) or three months (18.8%, 35/186) and allowed to defrost in the refrigerator (50.0%, 93/186) or at room temperature (44.6%, 83/186), while the remaining 5.4% of respondents defrosted it using the microwave or hot water. About half of the interviewees prepared the daily rations in advance and stored them cooked in the fridge (21.6%, 48/222) or in the freezer (30.2%, 67/222), while the other half (48.2%, 107/222) prepared the meals one by one. As for managing leftovers, 48.2% (107/222) of the participants tossed them in the trash, 40.5% (90/222) stored them until the following meal, and 11.3% (25/222) left them in the bowl to be consumed later by the pet.

### 3.9. Raw Meat-Based Diets: Management, Purchase and Storage

Two hundred and thirty-six respondents fed their pets a raw diet. There were three main reasons behind their choice: the first was the belief that RMBDs are the most biologically appropriate diets for dogs and cats owing to their ancestors’ carnivorous nature (42.4%, 100/236); the second was their distrust of commercial pet food (25.0%, 59/236), the third regarded their pets’ health problems (24.6%, 58/236). A small minority chose it on a veterinarian’s advice (3.8%, 9/236) or because the pet refused to eat kibbles (2.5%, 6/236) or because the pet was weaned on this diet by the breeder (1.7%, 4/236). The most common RMBD type was the BARF diet (83.9%, 198/236) followed by the Paleo Diet (2.1%, 5/236), the Prey-Model Diet (1.3%, 3/236) and the Ultimate Diet (0.4%, 1/236); the remaining 12.3% owners (29/236) fed their pets raw meat products without following any particular diet theory.

About one third of the owners (36.9%, 87/236) trusted a veterinary nutritionist for the formulation of the diet, 5.9% (14/236) relied on their own veterinarian and 7.2% (17/236) on an online nutritionist consultant. The other half of the respondents instead opted for homemade recipes formulated according to indications in books (19.9%, 47/236), information available on internet (19.9%, 47/236) or their own expertise (10.2%, 24/236).

As regards the feeding schedule, most dogs were fed twice a day (68.6%, 162/236), while 17.8% (42/236) of the owners administered one meal per day and 13.6% (32/236) three or more. The quantity of food provided was generally weighed with a kitchen scale (91.1%, 215/236); alternatively, 7.6% (18/236) of the daily rations were defined by eye and 1.3% (3/236) not measured at all. Fish was included in the diet by 89% of the RMBD-feeders (210/236). Solid fats were used by 43.2% of the owners (102/236), the most common were lard (11%, 26/236), tallow (6.4%, 15/236) and butter (5.1%, 12/236). Meat and fish were purchased from the butcher or fishmonger (30.0%, 71/236), at the supermarket (20.8%, 49/236), on internet (7.6%, 18/236), or from any of these places (41.5%, 98/236).

Most respondents (85.6%, 202/236) bought fresh meat and fish to be frozen at home, while the minority purchased them already frozen (9.3%, 22/236) or bought and stored only fresh ingredients (5.1%, 12/236). Except for those who used only fresh ingredients, the meat was stored in the freezer for no longer than one month by 54.0% (121/224) of the participants and for no more than three months by 26.8% (60/224); 19.2% (43/224) stored it for more than three months. The food was defrosted in the refrigerator (52.7%, 118/224) or at room temperature (42.4%, 95/224), while the remaining 4.9% (11/224) of the respondents used the microwave or hot water. Almost half of the owners (45.5%, 102/224) reported having a separate freezer for storing the meat intended for their pets.

As for the sanitary precautions taken by the owners when handling raw meat, 30.5% (72/236) used gloves and 25.4% (60/236) prepared the pet’s diet on a different work table than the one used for human meals. Whether the diet was formulated by the pet owner or the veterinarian made no difference in the proportion of people using gloves (*p* = 0.2580) and different working tables (*p* = 0.3697) during preparation.

The daily rations were prepared in advance and then stored in the refrigerator or the freezer by 58.9% (139/236) of participants, while 41.1% (97/236) prepared the meals one by one.

Leftovers were generally included in the following meal (58.0%, 137/236) or tossed in the trash (37.3%, 88/236), while a small minority left them in the bowl at the animal’s disposal (4.7%, 11/236).

### 3.10. Use and Storage of Fish Oils and Vegetable Oils

Among the owners who chose a HCD or a RMBD for their pet, 62.2% (285/458) purchased omega-3 fatty acids supplements in the form of pearls or drops (31.7%, n/285), fish oil (19.7%, 140/285) or both (10.9%, n/285). The supplementation of omega-3 fatty acids was equally spread among HCD-feeders (64.4%, 143/222) and RMBD-feeders (60.2%, 142/236), as well as among cat (64.9%, 24/37) and dog (62.0%, 261/421) owners.

The most commonly used fish oils were salmon oil (75.7%, 106/140), cod liver oil (20.0%, 28/140) and krill oil (14.3%, 20/140); two people used a seaweed-derived oil (1.4%).

Fish oil was stored in the refrigerator by 61.4% (86/140) and at room temperature by 38.6% of the respondents (54/140); such percentage did not vary much between those who formulated the diet themselves (41.5%) and those who relied on a veterinarian (37.3%). Over half of the owners (54.3%, 76/140) used the fish oil no later than one month after first opening and 18.6% (26/140) no later than two months; almost one third of participants (27.1%, 38/140) used it for more than two months. The fish oil packaging was transparent in 22.1% of cases (31/236). The fish oil was generally not exposed to light (97% of cases, 136/140) and temperatures above 30 °C (92.1%, 129/140).

The majority of respondents (69.2%, 317/458) used at least one vegetable oil, with a greater diffusion among HCD-feeders (80.6%, 179/222) than RMBD-feeders (58.5%, 138/236). Owners who relied on a veterinarian for the formulation of the diet (74.4%, 212/285) were more likely to use vegetable oils than those who formulated the diet by themselves (60.7%, 105/173) (*p* = 0.0029).

The most common vegetable oil types were olive oil (*n* = 157), sunflower oil (*n* = 130), coconut oil (*n* = 117), flaxseed oil (*n* = 106) and corn oil (*n* = 58). Vegetable oils were stored at room temperature by 79.8% of the respondents (253/317) and in the refrigerator by 20.2% (64/317). Most owners (77.9%, 247/317) used the vegetable oil no later than two months after opening; the remaining 22.1% (70/317) used it for more than two months. The vegetable oil packaging was transparent in 35.0% of cases (111/317). The vegetable oil was generally unexposed to light (95.6%, 303/317) and high temperatures (87.1%, 276/317).

## 4. Discussion

To the best of the authors’ knowledge, domestic pet food storage practices have not yet been considered in literature. This survey was intended to disclose how owners manage food for their pets from purchase to disposal and to highlight certain crucial points that can lead to food spoiling and unhygienic practices at home.

### 4.1. Diet Choice for Dogs and Cats and Its Relationship to Demographics

This survey provided evidence that the most common diet type used for both dogs and cats was kibble, which was fed either alone or in combination with canned food, and this finding is consistent with the latest official report drafted in Italy [16]. The percentage of owners who chose to feed a homemade diet (27.2%) has increased substantially compared to previous surveys conducted in Western countries [17,18,19,20]. The choice of a raw diet has become markedly popular in recent years, and almost equal numbers of RMBD-feeders and HCD-feeders were found in this study. The different prevalence of alternative diets in canine and feline populations should be noted, however, given that homemade diets were very scarcely adopted for cats but amounted to almost one third of the recruited dogs’ regimens. This difference between the two species has been reported in a previous study [21] and can be explained by the fact that the flavor and texture preferences of individual cats are often influenced by early experience that can affect preferences throughout life [22]. Cats accustomed to a specific texture or type of food (i.e., moist, dry, semi-moist) may refuse foods with different features, so switching their diet from commercial to homemade may be more difficult than it is in dogs.

According to our findings, owner age and pet age were both correlated with the type of diet offered to the animal, even if some differences emerged between the two species.

In particular, the use of industrial diets decreased as the dog’s owner aged. The younger segments of the population were instead more at ease in buying commercial pet food, probably because it offers a practical, time-saving-time solution better suited to the lifestyle of their age brackets. Commercial pet food may also be more commonly viewed as the routine pet feeding solution by younger generations grown used to seeing well-established products on the market, as opposed to many members of older generations who still remember when pets were commonly fed leftovers or other food shunned by the family.

It is interesting to note that whereas over-65s relied more on HCDs than any other respondent group, they favored RMBDs less than any other. The preference for HCDs in the older age bracket probably reflects the fact that they have more time to prepare meals than young owners, who prefer the convenience of kibble for such reason. The reluctance of over-65s to follow the new RMBD trend is probably also due to the lower internet usage by this age bracket [23] and consequent less familiarity with new trends.

The lack of relationship between owner’s age and cat’s diet probably continued to depend on the strong influence that early experience has on a cat’s dietary preferences throughout life. As regards the influence of the pet’s age on dietary preferences, puppies have been mainly fed industrial dry foods: this could be due to the convenience and the perceived safety of using a well-balanced diet specifically formulated to the needs of growing pets. Conversely, canned foods and HCDs have been more commonly adopted for senior dogs, probably to deal with aging-related issues such as periodontal diseases and taste and smell decline while avoiding the consumption of firm-textured foods such as kibbles. The same reasons can be used to explain the wider diffusion of commercial wet diets among older cats.

### 4.2. Dry and Wet Pet Food-Based Diets

This survey found evidence of widespread use of grain-free and single-protein pet foods. The use of grain-free diets has gained great popularity in the past few years and this has in part been encouraged by pet food companies, which have been releasing more and more high protein “ancestral diets”, even if there is no scientific evidence to support that feeding a grain-free diet is healthier than feeding its grain inclusive counterpart [24]. Although single-protein diets were originally formulated to diagnose and support specific food adverse reactions [25], they are commonly administered to healthy pets even if there is no evidence of extra benefits than those offered by standard maintenance foods. Moreover, one in four kibble feeders was unable to say whether the product they used was single-protein or not, meaning that a significant number of consumers was unaware of the significance and/or practicality of this feature that is increasingly emphasized on packaging.

As for dry pet food preservation, only a minority of owners stocked up kibble in large quantities at home, since the majority preferred to buy only one or two packages at a time. Half of the purchased pet food bags purchased weighed more than 5 kg, however, and in almost two out of three cases the animal took at least four weeks to consume an entire package. The long-term use of opened pet food packages (especially in the case of dogs, who usually took more time than cats to consume a package) combined with the owner’s belief that the quality of the kibbles remains unaltered for several months after opening increase the importance of adequate food preservation. Overall, it can be said that almost every owner was diligent in closing the original packaging, thus preventing environmental contamination and reducing air exposure. The habit of storing kibble in a dedicated container was quite widespread, with bins in plastic much more common than those in tin. Between the two, plastic is probably the better material for storing kibble. Metals can accelerate the oxidative process on the food substrates they come in contact with [26]. The characteristics of the original packaging also played a non-secondary role since it was still used after the opening to contain all or some kibbles by eight out of ten owners. Although modern multi-material packaging is practical and ensures the best product preservation, cardboard packaging appears to be still quite common, even if it is not the most appropriate choice to protect kibbles from ambient humidity [27]. The owners interviewed also reported taking care to avoid exposure to light and heat, but nearly one out of two kept some dry pet food in the kitchen, usually one of the warmest rooms in the house. One out of four owners admitted that they could not be sure that the food was never exposed to temperatures above 30 °C in the summer. All things considered, exposure to light seemed the best managed critical point in kibble storage, whereas exposure to humidity and heat appeared less under their control.

Palatability is related to sensory properties such as flavor, aroma and texture, which can be strongly influenced by storage condition [28]. Even when storage conditions are satisfactory, the deterioration process progresses inevitably from when the bags are opened. Over time, pet food deterioration can lead to negative palatability [5], as the organoleptic qualities are inevitably affected, and this is probably perceived earlier by the animal, whose senses are sharper than a human’s. It is therefore only reasonable that one third of the owners thought their pet was more attracted to kibble from newly-opened bags than kibble from sacks open for a long time. Although this result depended on owners’ individual sensitivity to alterations in their pet’s feeding behavior, it was even more consistent for the feline population, in which half of the cats was reported to prefer dry pet food from newly-opened bags.

Anomalies in kibble such as unpleasant odors and the presence of insects were rare but not absent and were more frequently noticed at the opening of the packages than during subsequent use. Dry pet food provides a suitable substrate for the reproduction of storage mites [29,30,31] and environmental conditions were clearly proven to be a major factor involved in their contamination: high temperature (25–30 °C) and humidity (80%) were shown to foster the growth of this pest [29,31]. Storage mites are also considered important allergens for dogs with atopic dermatitis [32]; therefore, the importance of storing kibble properly should be stressed to the owners, especially when the products are intended for animals with sensitivities.

Only a small minority of those interviewed adopted a canned food-based diet, probably due also to the higher maintenance cost of this type of diet, especially for large and giant size dogs. Almost one in three owners used single-dose pet food packages, raising no storage issues after opening. Among the others, the preservation of wet pet foods can be considered satisfactory as cans were generally lidded, stored in the refrigerator for a maximum of one day and heated before being fed to the pet. It can be argued that the storage of wet pet food presents fewer after-opening criticalities due to the small sizes of the packages that allow the rapid consumption of the products; however, given the high water content, it should be noted that canned food provides an optimal substrate for the growth of spoilage microorganisms [33], thus the enforcement of proper preservation practices remains essential.

### 4.3. The Owner’s Opinion on the Use of Preservatives in Commercial Pet Food

The results from the Likert scale questions yielded important information on the owners’ attitude to additives: the inclusion of such substances in pet food was deemed unnecessary and even unhealthy by the majority of the respondents, particularly the older age brackets. This was probably due to the perceived lack of “naturalness” of preservatives, which were identified as predominantly synthetic by the majority of the sample. Such results are in line with those from surveys on the use of preservatives in food intended for human consumption, which show that consumers have little trust in artificial food additives [34,35]. However, Shim et al. [36] showed that the consumer’s awareness and knowledge of the use of preservatives greatly influence the safety perceived: whenever appropriate information was provided, the percentage of people who expressed a positive opinion of them more than doubled. Pet food manufacturers should therefore aim at providing owners with targeted information on the usefulness and safety of additives in order for them to develop conscious knowledge of the question. Veterinarians should also help consumers build a solid and correct opinion on the use of preservatives in pet food. This survey also showed that veterinarians were not the owners’ preferred source of information on pet feeding, however, and that neither did they regularly seek a veterinarian’s advice on the type of diet and the feeding quantity for their pet. Internet has become a very popular tool, not only for purchasing pet food, used for the purpose by a quarter of the interviewees, but also for acquiring information: almost one in five participants relied on the web for the choice of the dry diet, and almost one in three for the wet diet.

### 4.4. Home-Cooked and Raw Meat-Based Diets

The study suggests that Italian owners chose to feed their pets a HCD mainly due to their distrust of commercial food or to better deal with the pathological conditions of their pets. Surveys conducted previously have reported the same two main reasons for choosing this type of diet [18,37]. The same reasons were also stated by RMBD-feeders, even if their very first motivation was the belief that raw food is more biologically-appropriate for dogs and cats, and this result confirms that of a previous study carried out in Italy [38]. The abovementioned scarce appreciation of additives may be blamed as one of the reasons that eventually leads to the lack of trust in industrial pet food and to the demand for alternative “more natural” diets [39].

Fewer RMBD-feeders (i.e., one in two) than HCD-feeders (i.e., three in four) relied on a professional figure for the formulation of their pets’ diets. The strong tendency of RMBDs supporters to formulate pet diets by themselves, especially with support from internet and books, instead of turning to a veterinarian or a nutrition-trained expert, has been disclosed previously [38,40]. The frequency with which do-it-yourself recipes and ready-to-eat meals are linked to an inadequate intake of many nutrients [41,42,43], such as minerals like calcium and phosphorus and vitamins like vitamin D in RMBDs, above all [44] is well worth noting. The need to consult a veterinary nutritionist when formulating a homemade diet in order to avoid nutritional imbalances that can lead to serious long-term health consequences should always be stressed [38,41,42,43].

Homemade diets were generally administered as two meals per day; the number of owners who preferred to feed the animal a single daily meal was much higher among RMBD-feeders (i.e., three times the HCD-feeders) however, probably due to the fact that less frequent and larger meals resemble the natural eating behavior of the dog’s ancestors.

Only a small percentage of respondents reported always using fresh ingredients for the preparation of their pets’ diets, while freezing was the most common method of meat preservation. When frozen, meat was preserved for only a very short time before use, as is usually the case of meat for human consumption [45]: the vast majority did not leave the meat in the freezer for more than three months, a limit that meets the guidelines regardless of type of meat [46]. Many owners reported defrosting meat at room temperature, whereas a temperature between 5 °C and 7 °C is considered optimal for safe thawing, and higher temperatures have been proven to promote faster bacterial growth [46]. Moreover, some studies revealed that operational temperatures in domestic refrigerators are often higher than 5 °C and sometimes even 10 °C, which may lead to faster food degradation and bacterial growth [45,47]. Therefore, as demonstrated elsewhere [48], storing pet food in the refrigerator or freezer may still pose some concerns, especially with RMBDs, whose ingredients are administered uncooked regardless of thawing method.

Managing a RMBD involves major microbiological risks linked to handling raw meat, which is often contaminated by pathogenic bacteria, some of which with zoonotic potential [49]. If not managed properly, RMBDs can pose threats to animal and human health. Bacterial foodborne illness in pets and related human infections have already been reported [50,51,52]. However, many owners seem to underestimate the microbiological risk posed by RMBD preparation [38] and this survey showed that few respondents followed safe food handling recommendations, like working on dedicated surfaces (i.e., one in four) or wearing gloves (i.e., less than one in three); a recent worldwide internet-based survey involving more than 16 thousand respondents reported a similar result, namely that the majority of the RMBD-feeders interviewed used the same place and utensils regardless of whether the raw meat was intended for their pets or their own consumption [50]. Moreover, almost half of the interviewed RMBD-feeders regularly bought frozen ingredients online, and a recent study proved that these products may be already highly contaminated at delivery and spoil very rapidly, especially if not kept at lower refrigeration temperatures [48]. Given the higher risk rate, owners who choose to feed RMBDs should take the appropriate precautions to protect human and animal health both when handling and storing meat. It is also essential that veterinarians inform pet owners and retail employees of the hazards of feeding and handling raw food [52].

### 4.5. Use and Storage of Fish Oils and Vegetable Oils

The HCD and RMBD formulations investigated often included fish oils and vegetable oils. These products are at great risk of deterioration during storage due to their high content of polyunsaturated fatty acids omega-6 and omega-3 [53]. Especially fish oils are notably susceptible to spoilage because their oxidation rate is significantly higher than that of other oils [54], and increasing storage temperatures has been shown to significantly accelerate the oxidation process [55]. However, more than one in three respondents kept fish oils at room temperature and such percentage did not vary much between those who relied on a veterinarian and those who formulated the diet themselves. The proper storage of oils should not be overlooked and, when prescribing the diet, the veterinarian should always make sure the owner gets the right instructions in order to preserve the pet’s health and appetite. Especially in summer, fish oils should be stored in the refrigerator at +4 °C and their consumption is considered acceptable up to 90 days of opening the bottle [55]. Vegetable oils rich in PUFAs should also be stored at low temperatures in order to slow down the oxidation process [56].

### 4.6. Management of Leftovers

Lastly, a substantial difference emerged among the four types of diet regarding the management of leftovers. The dry diet was the one with the lowest rate of food waste, probably thanks to the slower perishability of the product. Among the alternative diets, owners who fed RMBDs were more attentive to this aspect than those who feed HCDs: this could be due to the fact that a raw meat-based meal is on average more expensive than a home-cooked meal because it contains more amounts of expensive foods (like meat and fish) and fewer amounts of cheaper foods (like rice, pasta or other sources of carbohydrates). Consequently, owners may be more reluctant to throw the raw meat leftovers in the trash and prefer to keep them refrigerated until the next meal. More conscientious management of leftovers by dog and cat owners may be the merit of growing global awareness and commitment to the fight against food waste: given the large amount of meat products included in home-prepared diets for pets, this kind of waste and its environmental impact cannot be deemed negligible [57].

### 4.7. Limits of the Study

Certain limitations in this study should be considered in order to better interpret its results. First of all, the limit imposed by language made the sample representative only of Italian pet owners. Secondly, the survey was shared only on one social media and the respondents were self-selected, so there may have been a sampling bias (e.g., age). Given the length of the survey, it is possible that only the most conscientious owners took the time to fill it out, and these people may be more concerned with storage issues than average. In order to gain more comprehensive knowledge on the subject, additional studies should investigate the storage practices of owners in different countries. It might also be useful to examine the peculiarity of every single diet (i.e., commercial, HCDs, RMBDs) in greater detail with ad hoc questionnaires. Finally, laboratory tests should be conducted to verify the microbiological and chemical risks of pet food under different storage conditions.

## 5. Conclusions

In conclusion, kibble was the most common pet food adopted by the dog and cat owners involved in this study. Wide participation in the survey permitted the acquisition of evidence that the pet’s age affects the choice of diet in both species, whereas the owner’s age plays a role in the choice of the diet for dogs but not for cats.

Bearing this in mind, the survey also demonstrated that owners care about pet food preservation, and this was reflected in overall their good storage management despite the wide variability of practices and type of diet. Some critical issues emerged nonetheless, such as the possibility that dry pet foods and oils may be unintentionally exposed to high temperatures, in this way definitely increasing their risk of going rancid. The most delicate aspects regarding pet food storage should not be overlooked by veterinarians when questioned about proper diet management by pet owners regardless of the type adopted. Veterinarians should also provide precise instructions on storing highly perishable ingredients (e.g., fish oil) to those who formulate home-prepared diets.

Finally, many people showed a strong dislike for the inclusion of preservatives in commercial products, and this was especially evident for those in the older age brackets. Manufacturers should adopt more explicative labelling and advertising that emphasizes the importance of additives.

## Figures and Tables

**Table 1:** Demographics of survey respondents (*n* = 2221).

		Owners, *n* (%)
Gender	Male	281 (12.6)
	Female	1940 (87.4)
Age, years old	<18	10 (0.4)
	18–34	894 (40.3)
	35–44	551(24.8)
	45–54	467 (21.0)
	55–64	237 (10.7)
	>65	62 (2.8)
Geographical distribution	Northern Italy	1397 (62.9)
	Central Italy	491 (22.1)
	Southern Italy and Islands	301 (13.6)
	Other countries	32 (1.4)

**Table 2:** Characteristics of dogs enrolled in the study (*n* = 1545).

		Dogs, *n* (%)
Gender	Male	790 (51.2)
	Female	755 (48.8)
Neutering status	Neutered/spayed	595 (38.5)
	Intact	950 (61.5)
Age	<1 y.o. (puppy)	254 (17.1)
	1–7 y.o. (adult)	968 (65.1)
	>7 y.o. (senior)	265 (17.8)
Body condition (according to owner)	Underweight	70 (4.6)
	Normal weight	1317 (85.2)
	Overweight	156 (10.1)
Most represented breeds(8 out of 116)	Mongrel	302 (19.5)
	Border Collie	90 (5.8)
	Weimaraner	69 (4.5)
	Labrador Retriever	65 (4.2)
	Dachshund	62 (4.0)
	French bouledogue	62 (4.0)
	German shepherd	48 (3.1)
	Boxer	46 (3.0)

**Table 3:** Characteristics of cats enrolled in the study (*n* = 676).

		Cats, *n* (%)
Gender	Male	367 (54.3)
	Female	309 (46.7)
Neutering status	Neutered/spayed	591 (87.4)
	Intact	85 (12.6)
Age	<6 m.o. (kitten)	41 (6.1)
	6–24 m.o. (junior)	157 (23.5)
	2–10 y.o. (adult)	384 (57.3)
	>10 y.o. (senior)	88 (13.1)
Body condition (according to owner)	Underweight	27 (4.0)
	Normal weight	482 (71.3)
	Overweight	167 (24.7)
Most represented breeds(7 out of 24)	European Shorthair, no pedigree	517 (76.4)
	Maine Coon	44 (6.5)
	Scottish Fold	15 (2.2)
	Norwegian Forest Cat	14 (2.1)
	Ragdoll	10 (1.5)
	Siberian	10 (1.5)
	Persian	9 (1.3)

**Table 4:** Distribution of the types of diets offered to dogs and cats (***n***/%) based on owner’s and pet’s age.

		Dry Pet Food *n* (%)	Wet Pet Food *n* (%)	HCD *n* (%)	RMBDs *n* (%)
Species	Dog	1064 (68.8)	60 (3.9)	214 (13.8)	207 (13.4)
	Cat	618 (91.4)	21 (3.1)	8 (1.2)	29 (4.3)
Dog					
Owner’s age	<18	9 (100)	0 (0)	0 (0)	0 (0)
	18–34	482 (75.1)	15 (2.3)	61 (9.5)	84 (13.0)
	35–44	244 (66.6)	15 (3.9)	57 (15.8)	50 (13.7)
	45–54	197 (62.3)	19 (6.1)	55 (17.4)	45 (14.2)
	55–64	104 (63.0)	10 (6.1)	27 (16.4)	24 (14.5)
	>65	28 (59.6)	1 (2.2)	14 (29.7)	4 (8.5)
Dog’s age	<1 y.o.	209 (82.3)	3 (1.2)	21 (8.3)	21 (8.3)
	1–7 y.o.	650 (67.1)	40 (4.1)	134 (13.9)	144 (14.9)
	>7 y.o.	187 (62.7)	15 (5.0)	56 (18.8)	40 (13.4)
Cat					
Owner’s age	<18	1 (100)	0 (0)	0 (0)	0 (0)
	18–34	237 (94.0)	6 (2.4)	0 (0)	9 (3.6)
	35–44	170 (91.9)	4 (2.2)	3 (1.6)	8 (4.3)
	45–54	133 (88.1)	7 (4.6)	4 (2.7)	7 (4.6)
	55–64	63 (87.5)	3 (4.2)	1 (1.4)	5 (6.9)
	>65	14 (93.3)	1 (6.7)	0 (0)	0 (0)
Cat’s age	<6 m.o.	36 (87.8)	2 (4.9)	0 (0)	3 (7.3)
	6–24 m.o.	146 (93.0)	2 (1.3)	3 (1.9)	6 (3.8)
	2–10 y.o.	356 (92.7)	6 (1.6)	4 (1.0)	18 (4.7)
	>10 y.o.	74 (84.1)	11 (12.5)	1 (1.1)	2 (2.3)

## Data Availability

The data presented in this study are available on request from the corresponding author. The data are not publicly available due to privacy reasons.

## Supplementary Materials

The following are available online at https://www.mdpi.com/2076-2615/11/2/273/s1, File S1: original survey (English translation).
