# Supplementary material for: A Survey among Dog and Cat Owners on Pet Food Storage and Preservation in the Households
Source: Animals (Basel). 2021 Jan 21;11(2):273. doi: 10.3390/ani11020273 (PMC7911149; doi:10.3390/ani11020273)
Supplement: Supplementary file 1 [file animals-11-00273-s001.pdf]

Supplementary file 1, S1: Survey on pet food storage. Letters a,b,c,d refer to dry pet food, wet pet food, home-cooked diet and raw meat-based diet related questions, respectively.

1/8 – Owner data

1-Sex
- Male
- Female

2-Age
- <18
- 18-25
- 25-34
- 35-44
- 45-54
- 55-64
- >65

3-Province of residence

____

4-Animal-related job
- No
- Yes, veterinarian
- Yes, vet student
- Yes, dog trainer
- Yes, groomer

2/8 – Species

5-You are the owner of a
- Dog
- Cat

3.dog/8 – Dog signalment

6-Breed
- Mongrel
- Airedale terrier
- Akita Inu
- Great Dane
- Alaskan Malamute
- American Staffordshire terrier
- Australian Cattle Dog
- Australian Shepherd
- Basenji
- Beagle
- Bobtail
- Border Collie
- Boston terrier

- Boxer
- Bull terrier
- Bullmastiff
- Cavalier King Charles Spaniel
- Chihuahua
- Chow Chow
- Dobermann
- Dogue de Bordeaux
- Fox terrier
- Golden retriever
- Greyhound
- Jack Russell Terrier
- Labrador Retriever
- Parson Russell Terrier
- Rottweiler
- Schnauzer
- Shetland Sheepdog
- Shiba Inu
- Shih Tzu
- Siberian Husky
- Weimaraner
- Welsh Corgi Cardigan
- Welsh Corgi Pembroke
- West Highland White Terrier
- Whippet
- Yorkshire Terrier
- Other

7-If you have selected "other" in question 6, please specify your dog breed here

____

8-Sex

- Intact male
- Neutered male
- Intact female
- Spayed female

9-Date of birth

____

10-Weight

____

11-Size

- Small (<10kg)
- Medium (10-25kg)
- Maxi (25-45kg)
- Giant (>45kg)

12-How do you evaluate the body condition of your dog?

- Ideal
- Underweight
- Overweight
- Obese

13-Where does your dog live most of the time?

- In the house
- In the garden
- Both

3.cat/8 – Cat signalment

14-Breed

- European shorthair (no pedigree)
- American Curl
- American shorthair
- Bombay
- British shorthair
- Cornish Rex
- Devon Rex
- Egyptian Mau
- Exotic Shorthair
- Foreign White
- Havana Brown
- Korat
- Maine Coon
- Manx
- Munchkin
- Ocicat
- Peterbald
- Ragdoll
- Scottish Fold
- Sphynx
- Toyger
- Other

15-If you have selected “other” in question 6, please specify your cat breed here

____

16-Sex

- Intact male
- Neutered male
- Intact female
- Spayed female

17-Date of birth

____

18-Weight

____

19-How do you judge the body condition of your cat?
- Ideal
- Underweight
- Overweight
- Obese

20-Where does your cat live most of the time?
- In the house
- In the garden
- Both

4/8 – Type of diet

21- What do you feed your pet at the moment?
- Only dry food
- Dry + wet food
- Dry + homemade food
- Dry + wet + homemade food
- Only wet food
- Wet + homemade food
- Home-cooked food
- RMBDs

5a/8 – Dry diet

22-How many times do you feed your pet daily?
- 1
- 2
- 3
- 4 or more
- Ad libitum

23-How do you establish the daily amount of kibble you provide?
- According to the feeding instructions on the package
- According to the feeding instructions by the veterinarian
- According to my personal experience

24-How do you quantify the daily dose of kibble?
- By sight
- With a scale
- With a measuring cup
- I don't measure it

25-Where do you buy the pet food?
- At the supermarket
- In a pet store chain
- At the local pet shop
- On the internet
- From a door-to-door salesman

26-How many packages do you usually buy?
- 1

- 2
- 3
- 4
- 5 or more

6a/8 – Dry diet selection and management

27-The pet food you are feeding at the moment is for:
- Puppies
- Adults
- Seniors
- For all ages
- Unspecified on the package

28-The pet food you are feeding at the moment is:
- Dietetic
- Standard maintenance pet food
- Grain-free
- Calorie-restricted
- Breed-specific
- Vegetarian
- Vegan
- Other ________________________________

29-If you are feeding a dietetic pet food, what disease is it for?
- Obesity
- Gastrointestinal
- Dermatological
- Renal
- Hepatic
- Cardiac
- Other ________________________________

30-Has the pet food you are feeding a single-protein formulation?
- Yes
- No
- I don't know

31-What is the weight of the package you are using?
- 200-450g
- 500-900g
- 1-2kg
- 3-4 kg
- 5-6 kg
- 7-10kg
- 11-15 kg
- More than 15 kg
- I purchased bulk kibbles

32-How long does it take your pet to consume an entire package?
- Less than a week

- About one week
- About two weeks
- About three weeks
- About four weeks
- 1-2 months
- 3-4 months
- More than 4 months

33-The pet food you are feeding is:

- For small sized dogs
- For medium sized dogs
- For maxi sized dogs
- For giant sized dogs
- For all sized
- Unspecified on the package
- For cats

34-Optional: write the brand and the name of the dry food you are feeding

____

35-Why did you choose the pet food you are currently feeding?

- The veterinarian recommended it to me
- The breeder recommended it to me
- The pet shop assistant recommended it to me
- Friends/relatives recommended it to me
- I chose it in light of internet researches
- I chose it in light of my personal experience
- Other ________________________________

7a/8 – Dry food storage

36-How do you store the pet food?

- In the original package
- In another container
- Some is kept in the original package and some is moved to another container

37-Is the package made of cardboard?

- Yes
- No

38-How do you close the original package?

- I don't use the original package
- I don't close it
- I roll the edges of the bag
- I use the included resealable zip lock
- I use clothespins, adhesive tape and elastic bands
- Other ________________________________

39-What material is the alternative container made of?

- I don't use another container
- Plastic bin
- Tin bin

- Glass container
- Nylon bags
- I use an automatic pet feeder
- Other _______________________________

40-Where do you store the pet food?
- In the kitchen
- In the closet
- In other rooms
- Outside the house, in a closed environment
- Outside the house, outdoor
- Other _______________________________

41-Where do you keep the pet food bag?
- Inside a piece of furniture
- In another container
- On the ground, not in direct contact with the floor
- On the ground, in direct contact with the floor

42-Is the pet food package or container exposed to light?
- No, it's in the dark
- No, it's in a lit room but not directly exposed to light
- Yes, it's in a lit room

43-Is there a heat source near the pet food package or container?
- No
- Yes, the radiator
- Yes, the stove
- Yes, the fireplace
- Yes, sunlight
- Other _______________________________

44-Can the temperature of the room where the pet food is stored exceed 30°C?
- Yes
- No
- I don't know

45-Once the package has been opened, how long do you feel confident in feeding your pet that food before considering it spoiled?
- < 1 month
- 1-2 months
- 3-4 months
- 5-6 months
- > 6 months

46-Based on your experience, does your pet find the kibbles from the newly opened package more palatable than those from a long-opened package?
- Yes, always
- Yes, sometimes
- No, never
- I don't know

47-When opening a new package of feed (unexpired), has it ever happened to you to smell anomalous/unpleasant odors?

- No, never
- Yes, once
- Yes, more than once

48-Has it ever happened to you that abnormal/unpleasant odors developed during the regular use of the pet food?

- No, never
- Yes, once
- Yes, more than once

49-Did you ever find insects or insect larvae in the pet food at the opening of an unexpired food package?

- No, never
- Yes, once
- Yes, more than once

50-Did you ever find insects or insect larvae inside the package during the regular use of the food?

- No, never
- Yes, once
- Yes, more than once

51-Do you feed pet food even after the expiration date printed on the package?

- No, never
- Yes, sometimes
- I don't know, I don't pay attention to the expiration date

52-What do you do when you find uneaten kibbles left in the bowl after the meal?

- I leave them in the bowl so my pet can eat them later
- I store them in the fridge until the next meal
- I store them at room temperature until the next meal
- I toss them in the trash

5b/8 – Wet diet

53-How many times do you feed your pet every day?

- 1
- 2
- 3
- 4 or more
- Ad libitum

54-How do you establish the daily amount of wet food you provide?

- According to the indications of the package
- According to the indications of the veterinarian
- According to my personal experience

55-Where do you buy the pet food?

- At the supermarket
- In a pet store chain

- At the local pet shop
- On the internet
- From a door-to-door salesman

6b/8 – Wet diet selection and management

56-The pet food you are feeding at the moment is for:
- Puppies
- Adults
- Seniors
- For all ages
- Unspecified on the package

57-The pet food you are feeding at the moment is:
- Dietetic
- Standard maintenance pet food
- Grain-free
- Calorie-restricted
- Breed-specific
- Vegetarian
- Vegan
- Other ________________________________

58- If you are feeding a dietetic pet food, what disease is it for?
- Obesity
- Gastrointestinal
- Dermatological
- Renal
- Hepatic
- Cardiac
- Other ________________________________

59-Has the pet food you are feeding is a single-protein formulation?
- Yes
- No
- I don't know

60-What is the weight of the package you are feeding?
- 50-90g
- 100-390g
- 400-690g
- 700-900g
- 1-2 kg
- 7-10kg
- More than 2 kg

61-The pet food you are feeding is:
- For small sized dogs
- For medium sized dogs
- For maxi sized dogs
- For giant sized dogs

- For all sizes
- Unspecified on the package
- For cats

62-Optional: write the brand and the name of the wet food you are using

____

63-Why did you choose the pet food you are currently feeding?

- The veterinarian recommended it to me
- The breeder recommended it to me
- The pet shop assistant recommended it to me
- Friends/relatives recommended it to me
- I chose it in light of internet researches
- I chose it in light of my personal experience
- Other ________________________________

7b/8 – Wet food storage

64-Which type of wet food packs do you purchase most often?

- Pouches
- Aluminum trays
- Rigid cans

65-Can the temperature of the room where the pet food is stored exceed 30°C?

- Yes
- No
- I don't know

66-Where do you keep the wet food pack once opened?

- At room temperature
- In the refrigerator
- I don't keep it because I use single-dose packs

67-How do you store the wet food pack once opened?

- Without closing it
- I use plastic wrap, tinfoil, clothespins
- Inside a plastic bag
- I remove the original packaging and store the remaining food in an airtight container
- I do not keep it because I use single-dose packs
- Other ________________________________

68-How long do you keep the wet pack once opened?

- 1 day
- 2 days
- 3 days
- 4 days
- More than 4 days
- I don't keep it because I use single-dose packs

69-Do you warm the wet food before feeding it to your pet after it has been stored it in the refrigerator?

- No, I don't

- Yes, I heat it up
- I do not keep the opened wet pack in the refrigerator
- I do not keep the wet pack because I use single-dose packs

70-Do you feed wet food even after the expiry date printed on the package?

- I never cared about the expiration date
- No, never
- Yes, it happened

71-What do you do when you find leftovers in the bowl after the meal?

- I leave them in the bowl so my pet can eat them later
- I store them in the fridge until the next meal
- I store them at room temperature until the next meal
- I toss them in the trash

8a+b/8 – Opinions on preservatives

72-How much do you agree with the following statement: the use of preservatives in pet food is necessary for the optimal conservation of the product

- Strongly agree
- Fairly agree
- Neither agree nor disagree
- Quite disagree
- Strongly disagree

73-How much do you agree with the following statement: the presence of preservatives in pet food could be harmful for pets health

- Strongly agree
- Fairly agree
- Neither agree nor disagree
- Quite disagree
- Strongly disagree

74-In your opinion, the preservatives in pet food are:

- All chemists
- Many chemical and few natural
- Half chemical and half natural
- Few chemical and many natural
- All natural

5c/8 – Home-cooked diet

75-Why did you choose to feed your pet a home-cooked diet?

- I have no trust in commercial pet food
- My pet refuses kibble
- The vet recommended it to me
- It was motivated by my pet's health problems
- Other ________________________________

76-Who formulated your pet's diet?

- I did, based on my experience
- I did, based on internet researches

- I did, following the instructions reported in books on the subject
- My veterinarian
- An online nutritionist
- A veterinary nutritionist who visited my pet

77-How many meals is your pet fed daily?
- I always leave the bowl full and my pet eats when he wants
- 1 meal a day
- 2 meals a day
- 3 meals a day
- 4 or more meals a day

78-How do you quantify the amount of food you feed your pet?
- By eye
- With a kitchen scale
- I do not measure it

79-Is fish a part of your pet's diet?
- No, I only use meat
- Yes, I use fish sometimes
- Yes, I use only fish and no meat

80-Do you include solid fats in your pet's diet?
- No
- Yes, tallow
- Yes, lard
- Yes, butter
- Yes, lard
- Other ________________________________

81-Where do you buy meat/fish for your pet?
- At the supermarket
- From the butcher’s/fishmonger’s
- On the internet
- At the slaughterhouse
- In any of these places

6c/8 – HCD food storage

82-Do you store meat/fish for your pet’s diet in the freezer?
- Yes, always
- Yes, sometimes
- No, I only use fresh ingredients
- I buy products already frozen

83-How long do you keep frozen meat in the freezer?
- Up to a maximum of 1 month
- Up to a maximum of 3 months
- Up to a maximum of 9 months
- Up to a maximum of 1 year
- As long as I want, it never spoils in the freezer
- I use fresh meat only

84-How do you defrost frozen meat?

- I defrost it in the fridge
- I defrost it at room temperature
- I defrost it using the microwave
- I defrost it in hot water
- I only use fresh meat
- Other ________________________________

85-How do you arrange the preparation of your pet's meals?

- I prepare them from time to time
- I prepare several portions at once (e.g. for the whole week) and keep them in the refrigerator
- I prepare several portions at once (e.g. for the whole week) and store them in the freezer

86-How long do you keep the portions already prepared?

- I do not prepare portions in advance
- 3 days maximum
- 5 days maximum
- 7 days maximum
- 10 days maximum
- More than 10 days

87--What do you do when you find leftovers in the bowl after the meal?

- I leave them in the bowl so my pet can eat them later
- I store them in the fridge until the next meal
- I store them at room temperature until the next meal
- I toss them in the trash

5d/8 – Raw meat-based diets

88-Why did you choose to feed your pet a raw diet?

- I have no trust in commercial food
- My pet refuses kibble
- The vet recommended it to me
- It is a matter of principle: the dog descends from the wolf, the cat is a carnivore, it is right that they eat raw meat
- It was motivated by my pet's health problems
- Other ________________________________

89-What kind of raw diet did you choose for your pet?

- Barf
- Volhard
- Paleo Diet
- Ultimate Diet
- Prey Model Diet
- I feed my animal raw meat but without following the directions of any particular diet
- Other _______________________________

90-Who formulated your pet's diet?
- I did, based on my experience
- I did, based on internet researches
- I did, following the instructions reported in books on the subject
- My veterinarian
- An online nutritionist
- A veterinary nutritionist who visited my pet

91-How many meals is your pet fed daily?
- I always leave the bowl full and he eats when he wants
- 1 meal a day
- 2 meals a day
- 3 meals a day
- 4 or more meals a day

92-How do you quantify the amount of food to give to your pet?
- By eye
- With the kitchen scale
- I do not measure it

93-Is fish a part of your pet's diet?
- No, I only use meat
- Yes, I sometimes use fish
- Yes, I use fish only and no meat

94-Do you include solid fats in your pet's diet?
- No
- Yes, tallow
- Yes, lard
- Yes, butter
- Yes, lard
- Other ________________________________

95-Where do you buy meat/fish for your pet?
- At the supermarket
- From the butcher’s/fishmonger’s
- On the internet
- At the slaughterhouse
- In any of these places

96-Is the work plan where you prepare the meal for your pet the same where you prepare the food for yourself and your family?
- Yes
- No

97-Do you use gloves to prepare your pet's meal?
- Yes
- No

6c/8 – RMBDs food storage

98- Do you store meat/fish for your pet’s diet in the freezer?
- Yes, always

- Yes, sometimes
- No, I only use fresh ingredients
- I buy products already frozen

99-How long do you keep frozen meat in the freezer?

- Up to a maximum of 1 month
- Up to a maximum of 3 months
- Up to a maximum of 6 months
- Up to a maximum of 1 year
- As long as I want, it never goes bad in the freezer
- I only use fresh meat

100-How do you defrost frozen meat?

- I defrost it in the fridge
- I defrost it at room temperature
- I defrost it using the microwave
- I defrost it in hot water I only use fresh meat
- Other _______________________________

101-Where do you store the frozen meat for your pet?

- In a special freezer dedicated exclusively to his meat
- In the same freezer where I store other foods
- I only use fresh meat

102-How do you arrange the preparation of your pet's meals?

- I prepare food for my pet from time to time
- I prepare several portions at once (for example for the whole week) and keep them in the refrigerator
- I prepare several portions at once (for example for the whole week) and store them in the freezer

103-How long do you keep the portions already prepared?

- I do not prepare portions in advance
- 3 days maximum
- 5 days maximum
- 7 days maximum
- 10 days maximum
- More than 10 days

104--What do you do when you find leftovers in the bowl after the meal?

- I leave them in the bowl so my pet can eat them later
- I store them in the fridge until the next meal
- I store them at room temperature until the next meal
- I toss them in the trash

7c+d/8 – Omega-3 fatty acids

105-Do you use fish oils or capsule supplements as sources of omega-3 fatty acids for your pet?

- Yes, fish oil
- Yes, capsule supplements
- Yes, both

- No

106-What sources of omega-3 fatty acids do you include in your pet's diet?
- Salmon oil
- Cod liver oil
- Krill oil
- Algae oil
- Other ________________________________

107-How do you store fish oil once opened?
- At room temperature
- In the refrigerator

108-How long do you feed fish oil after opening the package?
- Less than 1 month
- 1-2 months
- 2-3 months
- 3-6 months
- More than 6 months

10- The fish oil packaging is:
- Dark
- Transparent

110-The place where you store fish oil is:
- Dark
- Lit, because it is in a lit room
- It is in a lighted room but protected from light

111-Is it possible for the oil to be exposed to temperatures above 30°C in the summer?
- Yes
- No
- I do not know

7c+d/8 – Vegetable oils

112-Do you include vegetable oils in your pet's diet?
- Yes
- No

113-Which vegetable oils do you include in your pet's diet?
- Borage oil
- Safflower oil
- Coconut oil
- Sunflower oil
- Linseed oil
- Corn oil
- Walnut oil
- Olive oil
- Soybean oil
- Other ________________________________

114-How do you keep the vegetable oil once opened?

- At room temperature
- In the refrigerator

115-How long do you feed the vegetable oil once the package is opened?

- Less than 1 month
- 1-2 months
- 2-3 months
- 3-6 months
- More than 6 months
- I don't use vegetable oils

116-The packaging of the vegetable oil is:

- Dark
- Transparent

117- The place where you store the vegetable oil is:

- Dark
- Lit, because it is in a lighted room
- It is in a lit room but protected from light

118-Is it possible for the oil to be exposed to temperatures above 30°C in the summer?

- Yes
- No
- I do not know
